# Excellent Response to Palliative Chemotherapy for Pleural Recurrence of Uterine Papillary Serous Carcinoma

**DOI:** 10.4021/wjon704w

**Published:** 2013-09-27

**Authors:** Shantanu Singh, David Mack, Dianne Dookhan, Juthika Jyotimallika

**Affiliations:** a Duke LPMP Medical Center, North Carolina, USA

**Keywords:** Uterine papillary serous carcinoma, Pleural fluid cytology, Pleural recurrence, Chemotherapy

## Abstract

Uterine papillary serous carcinoma (UPSC) is an aggressive variant of endometrial cancer. Though the majority of women with UPSC have high risk of recurrence, recurrence limited only to the pleural space has not been previously reported. It is also unusual for it to occur as late as 10 years after the initial treatment as it is usually very aggressive. There is scant information in literature on response to treatment in these patients. A 65-year-old African-American woman was treated for FIGO stage IIA UPSC with total abdominal hysterectomy, bilateral salpingo-oophorectomy, and pelvic lymphadenectomy with ^32^P catheter placement, followed by adjuvant external beam radiation. She relapsed 10 years later with right pleural effusion and pleural based nodules. Fluid cytology was consistent with UPSC. She was treated with 6 cycles of carboplatin and paclitaxel with excellent clinical and biochemical response. She continues to remain disease free 5 years later. To our knowledge, this is the first reported case of UPSC recurrence limited to pleura, and this is also the first reported case of UPSC recurrence without evidence of disease 5 years after palliative chemotherapy.

## Introduction

Uterine papillary serous carcinoma (UPSC) is an aggressive variant of endometrial cancer. Although it represents only about 10% of all endometrial cancers, it accounts for almost 40% of endometrial cancer related deaths [1]. The 5-year survival rate for all stages of UPSC is only 53% compared with 83% for endometrioid carcinoma [2]. Though the majority of women with UPSC have a high risk of recurrence [1], recurrence limited only to the pleural space has not been previously reported. It is also uncommon for it to occur 10 years after the initial treatment as UPSC is usually much more aggressive. This report is encouraging in that it provides a promising 5 year follow-up of the patient after completing palliative treatment, giving a glimmer of hope in this usually aggressive cancer.

## Case Report

**Figure 1:**
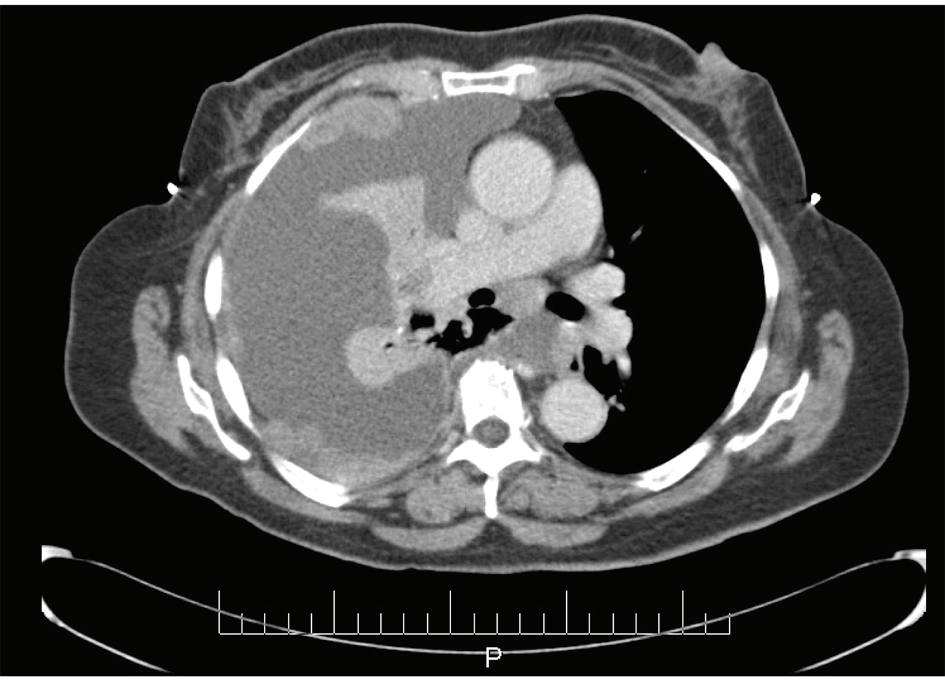
CT scan image demonstrating pleural effusion and nodularity.

In June 1997, a 65-year-old G9P009 African-American woman was treated for FIGO stage IIA UPSC with total abdominal hysterectomy, bilateral salpingo-oophorectomy, and pelvic lymphadenectomy with ^32^P catheter placement, followed by adjuvant external beam radiation. She was followed without clinical evidence of recurrence until December 2007, when she presented with shortness of breath of a few weeks duration. Her CA-125 level was elevated at 112 IU/mL. A chest x-ray showed a right side pleural effusion. A chest CT, in addition to confirming pleural effusion, also showed pleural-based nodules (Fig. 1). A diagnostic and therapeutic thoracentesis was done and 2 liters of serosanguineous fluid was removed.

**Figure 2:**
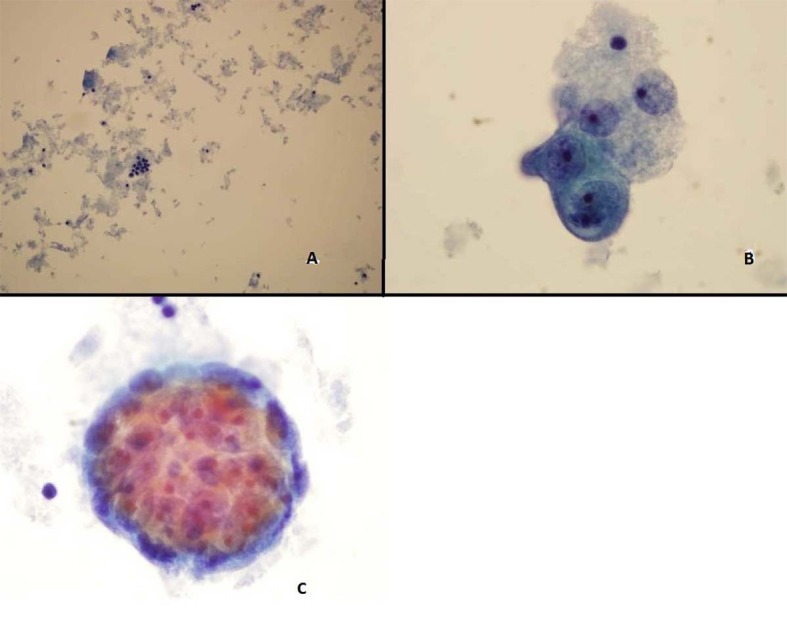
Pleural fluid Thin Prep demonstrating (A) Clusters of malignant epithelial cells in the background of necrotic cellular debris, 20 × (B) High power showing adenocarcinoma cells 100 × (C) 3-Dimensional clusters of adenocaricoma cells 100 ×

**Figure 3:**
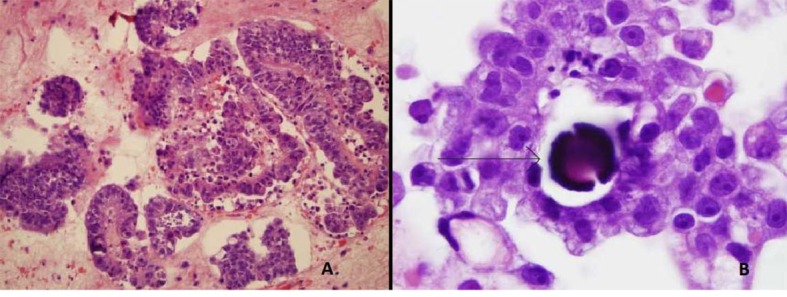
Pleural fluid cell block demonstrating (A) Papillary clusters of adenocarcinoma on H&E 20 ×. (B) Arrow showing Psammoma body on H&E 100 ×.

**Figure 4:**
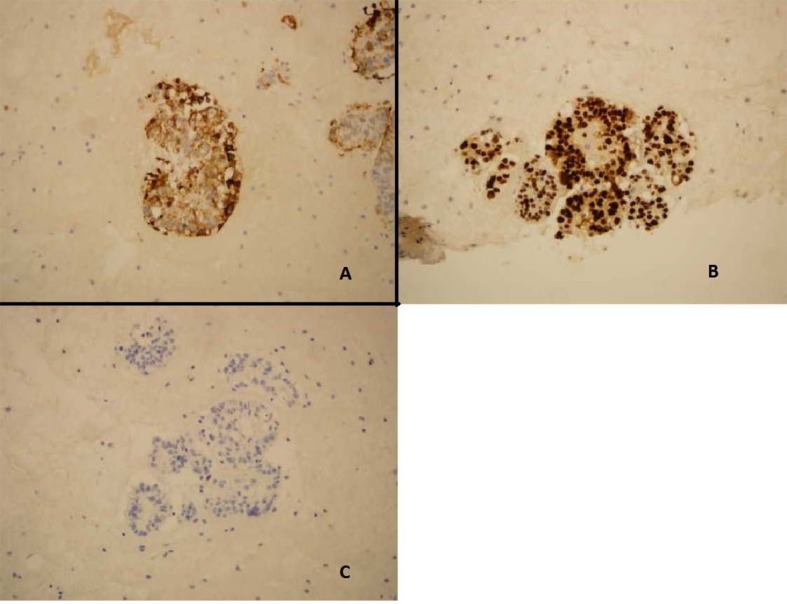
Pleural fluid cell block demonstrating (A) Immunostain positive for CK-7 (B) Immunostain positive for p53. (C) Immunostain negative for CK20.

ThinPrep of the pleural fluid demonstrated malignant cells consistent with adenocarcinoma (Fig. 2). H&E stain of the pleural fluid cell block demonstrated papillary clusters of adenocarcinoma with psammoma bodies, consistent with serous carcinoma (Fig. 3). Immunostains for p53 and CK7 were positive (Fig. 4A, B). CK20 (Fig. 4C), ER, PR, and WT-1 were negative. This pattern of immunoreactivity is consistent with serous carcinoma of uterine origin. Furthermore, immunostains for CK5, calretinin, TTF-1, and CEA, usually positive in thoracic adenocarcinomas, were all negative, making a primary lung or mesothelial neoplasm unlikely. Broncoscopy was nondiagnostic for pulmonary abnormalities, and CT of the abdomen and pelvis was negative for any evidence of extrathoracic disease.

**Figure 5:**
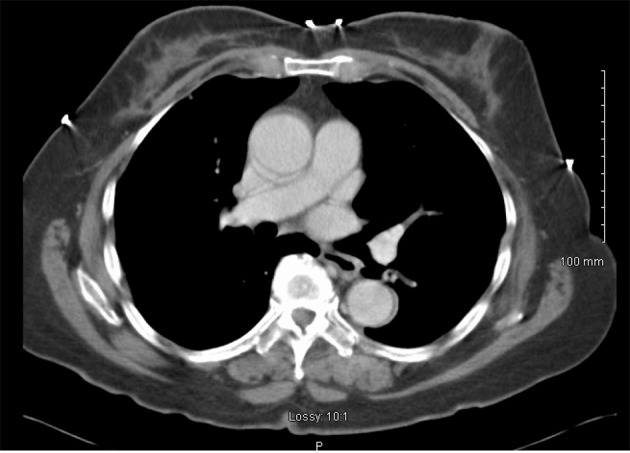
CT scan image after treatment demonstrating complete resolution of pleural effusion and nodularity.

She was treated with 6 cycles of carboplatin and paclitaxel at an AUC of 6 and 175 mg/m^2^ respectively, with excellent biochemical and clinical response. Her CA-125 level decreased to 3 IU/mL and follow-up CT chest showed complete resolution of pleural disease including the pleural nodules (Fig. 5). She continues to have surveillance visits and remains disease-free 5 years later. Her chest imaging remains normal and serum CA-125 level remains at 3 U/mL.

## Discussion

Endometrial cancer is now the most common gynecologic malignancy in Europe and North America [3]. These are a morphologically heterogeneous group of cancers. The most common type is endometrioid, which accounts for about 80% of all uterine cancers. UPSC, first described by Lauchlan [4] in 1981 and further described by Hendrickson et al [5] in 1982, accounts for only 5-10% of all uterine cancers. Bokhman [6] in 1983 divided endometrial cancers into two types: at present, Type I includes grade 1 and 2 endometrioid cancers, and Type II includes grade 3 endometrioid cancer and all non-endometrioid cancers (for example, UPSC, clear cell, mucinous).

Unlike in endometrioid cancers, women with UPSC tend to be of African ancestry, and are older and multiparous. Like other endometrial cancers, abnormal uterine bleeding is the most common presenting symptom. An office endometrial biopsy is sensitive but may not yield diagnosis in all cases [7]. UPSC has a complex papillary architecture that resembles serous carcinoma of the ovary; psammoma bodies are present in 60 percent of biopsy specimens. Diagnosis of UPSC is made if 10 percent or more of the tumor has a serous component. CA-125 may be elevated similar to serous epithelial ovarian cancers and may correlate with presence of extrauterine disease and poorer prognosis [8].

The initial management for the majority of women with UPSC is surgery [1]. As with other gynecologic malignancies, the goals of surgery are to obtain accurate staging and maximal cytoreduction. UPSC has a higher propensity for lymphovascular invasion and intraperitoneal and extra-abdominal spread than endometrioid cancers. At the time of presentation, approximately 60 to 70 percent of women with UPSC have disease spread outside of the uterus. Therefore, adjuvant treatment with a combination of chemotherapy, radiation therapy and brachytherapy is offered, although consensus on the type of adjuvant therapy for a specific stage is still evolving.

UPSC has the worst prognosis of all uterine cancers. Five year survival is only about 55 percent for UPSC while it is about 80% for endometrioid cancers. While UPSC comprises only about 5-10% of all uterine cancers, it causes almost 40% of all uterine cancer related deaths [9]. Recurrence occurs frequently and early. In one study, tumor recurred in 37% of the patients after a median of 15 months [10]. In another study, 67% of the patients had developed recurrence by 5 years [11]. Our case is unique as she did not develop recurrence until 10 years later. To our knowledge, this is longest interval to UPSC recurrence reported.

Our case is also unique in that this is the first case reported of UPSC recurrence limited to pleura. Of all the gynecologic malignancies, uterine cancer is the least likely to present with pleural disease [12]. In a study by Bouros et al [13], only 6 of the 90 uterine cancer patients who had pulmonary metastasis manifested a pleural effusion, and none of them were UPSC cases. The only reported case of pleural metastasis in UPSC had a malignant pleural effusion present at the time of initial diagnosis of UPSC [14].

Lastly, our case is unique that she remains disease-free 5 years after palliative chemotherapy. As agents useful in the management of ovarian serous carcinoma have evolved, the utility of chemotherapy in treatment of UPSC has been explored [1]. Zanotti et al first described administration of paclitaxel with or without either carboplatin or cisplatin in women with UPSC [1]. Other studies have also reported promising data on carboplatin and paclitaxel in UPSC [1]. Consistent with these studies, our case also documents excellent response to carboplatin and paclitaxel.

### Established facts

a). Uterine Papillary Serous Carcinoma is an aggressive variant of endometrial carcinoma; b). Recurrence occurs frequently and early; c). Prognosis is poor.

### Novel insights

a). This is the first reported case of UPSC recurrence limited only to pleura; b). This also is the first reported case of UPSC recurring as late as 10 years after the primary occurrence treatment; c). This also is the first reported case of still disease-free 5 years after the recurrence treatment; d). Diagnosis of this case was made solely by pleural fluid cytology highlighting its role.
